# Immature Responses to GABA in Fragile X Neurons Derived from Human Embryonic Stem Cells

**DOI:** 10.3389/fncel.2016.00121

**Published:** 2016-05-12

**Authors:** Michael Telias, Menahem Segal, Dalit Ben-Yosef

**Affiliations:** ^1^Wolfe PGD-Stem Cell Lab, Racine IVF Unit, Tel-Aviv Sourasky Medical Center, Lis Maternity HospitalTel-Aviv, Israel; ^2^Department of Cell and Developmental Biology, Sackler Medical School, Tel-Aviv UniversityTel-Aviv, Israel; ^3^Department of Neurobiology, The Weizmann Institute of ScienceRehovot, Israel

**Keywords:** Fragile X syndrome, GABA, neural development, human embryonic stem cells, *in-vitro* neural differentiation

## Abstract

Fragile X Syndrome (FXS) is the most common form of inherited cognitive disability. However, functional deficiencies in FX neurons have been described so far almost exclusively in animal models. In a recent study we found several functional deficits in FX neurons differentiated *in-vitro* from human embryonic stem cells (hESCs), including their inability to fire repetitive action potentials, and their lack of synaptic activity. Here, we investigated the responses of such neurons to pulse application of the neurotransmitter GABA. We found two distinct types of responses to GABA and sensitivity to the GABA-A receptor antagonist bicuculline; type 1 (mature) characterized by non-desensitized responses to GABA as well as a high sensitivity to bicuculline, and type 2 (immature) which are desensitized to GABA and insensitive to bicuculline. Type 1 responses were age-dependent and dominant in mature WT neurons. In contrast, FX neurons expressed primarily type 2 phenotype. Expression analysis of *GABA-A* receptor subunits demonstrated that this bias in human FX neurons was associated with a significant alteration in the expression pattern of the *GABA-A* receptor subunits α*2* and β*2*. Our results indicate that FMRP may play a role in the development of the GABAergic synapse during neurogenesis. This is the first demonstration of the lack of a mature response to GABA in human FX neurons and may explain the inappropriate synaptic functions in FXS.

## Introduction

Fragile X syndrome (FXS) is the most common form of inherited intellectual disability (Penagarikano et al., 2007; Bagni and Oostra, 2013). Individuals with FXS show cognitive disabilities, as well as high-comorbidity with autism and epilepsy. It is caused by developmentally regulated silencing of the *FMR1* gene, and absence of its encoded protein, FMRP (Willemsen et al., 2002). Loss of FMRP impairs neurogenesis of adult neural stem cells in mice (Luo et al., 2010; Guo et al., 2011) as well as embryonic neurogenesis of human pluripotent stem cells and neural precursor cells (Eiges et al., 2007; Adusei et al., 2010; Sheridan et al., 2011; Telias et al., 2013, 2015b).

In FX neurons obtained from *fmr1*^−∕−^ mice, the absence of the FMRP protein has been linked to an aberrant response to either the glutamate or the gamma-aminobutyric acid (GABA) neurotransmitter (Bear et al., 2004; Braat and Kooy, 2015). Several studies in *fmr1*^−∕−^ mice have demonstrated a link between the GABAergic inhibitory system and the impaired neuronal activity observed in FXS (Braat and Kooy, 2015), in which abnormal expression of GABAergic proteins in different brain areas and during different developmental stages has been shown (Adusei et al., 2010; Kratovac and Corbin, 2013). During development, neurons first respond to GABA with excitation and later switch to inhibition (Ben-Ari, 2015). It was later reported that such developmental switch is delayed in *fmr1*^−∕−^ mice (He et al., 2014). However, this hypothesis has not been tested in human FX neurons, mainly due to the lack of suitable research models. Therefore, analysis of the functional and molecular expression of the GABArgic synapse *in-vitro* in FX human neurons can shed new light on the involvement of inhibitory transmission in FXS.

We were able to derive FX human embryonic stem cells (hESCs) carrying the naturally occurring full mutation (>200 CGG repeats) at the *FMR1* locus, and demonstrated that *in-vitro* neural differentiation effectively produces neurons that gradually lose FMRP, and consequently demonstrate impaired neurogenesis (Telias et al., 2013). Recently, we have shown that this gradual downregulation of FMRP during neurogenesis until its complete absence in mature neurons leads to impaired electrophysiological properties, including lack of repetitive action potential firing, reduced neurotransmitter release and poor synaptogenesis (Telias et al., 2015a). In the present study we analyzed the functional response of these FX human neurons to GABA. Our results show that hESC neurons produce two types of responses to GABA, a mature (type 1) and an immature (type 2) response: in wild-type (WT) neurons, the immature responses are gradually replaced by the mature form, but in FX cells, type 2 responses are persistent. In addition, human FX neurons have increased RNA levels of *GABA-A*α*2* and reduced RNA levels of *GABA-A*β*2*. Taken together our results indicate that lack of FMRP may affect the composition of the GABA-A receptors and prevents their maturation.

## Materials and methods

### Human embryonic stem cells and *in-vitro* neural differentiation

The use of spare IVF-derived embryos diagnosed by Pre-implantation Genetic Diagnosis (PGD) for the derivation of hESCs was approved by the Israeli National Ethics Committee (7/04-043). Three Fragile X human embryonic stem cell (hESC) lines with >200 CGG repeats in the *FMR1* gene were studied: HEFX1 (male) and Lis_FX6 (male), derived at Tel-Aviv Sourasky Medical Center and SZFX6 (male), derived in Shaare Tzedek Medical Center. These three lines were entitled here systematically: FX1, FX2, and FX3, and were all fully characterized previously (Eiges et al., 2007; Telias et al., 2013). The hESC line HUES-13 (male; kindly provided by the Melton Lab, Harvard University), was previously shown by us to include <30 CGG repeats in the *FMR1* gene (Telias et al., 2013) and was used here as WT. hESCs were cultured on feeder layers of mitomycin C (Sigma)-inactivated mouse embryonic fibroblasts (MEF) in hES-medium supplemented with bFGF (R&D), as we previously described (Telias et al., 2013, 2015a). Before induction of *in-vitro* neural differentiation, hESCs were cultured on Matrigel (BD)-coated wells for two passages. The dual SMAD inhibition protocol was applied as we previously described (Telias et al., 2014, 2015a). Briefly, neural induction was achieved by gradually changing the medium from hES to N2 while adding dorsomorphin and SB431542 for 10 days; for neuronal induction the medium was changed to N2/B27 supplemented with BDNF, GDNF, ascorbic acid, dbcAMP and DAPT for 10 additional days. At day 20 cells were dissociated using Accutase (Life Tech.) and seeded on 13 mm glass coverslips pre-coated with 50 μg/ml Poly-D-Lysine and 20 μg/ml Laminin (Sigma) at a seeding density of ~1.0 × 10^5^ cells/cm^2^. From day 20, neurons were continuously grown in N2/B27 medium supplemented with 20 ng/ml BDNF, GDNF, and NT3, unless otherwise specified. Concentrations of reagents and growth factors used were as follows: 5 μM dorsomorphin (Stemgent), 10 μM SB431542 (Stemgent), 20 ng/ml BDNF (PeproTech), 20 ng/ml GDNF (PeproTech), 0.2 mM ascorbic acid (Sigma), 0.5 mM dbcAMP (Sigma), 10 μM DAPT (Tocris), 20 ng/ml NT3 (PeproTech). N2 medium was composed of DMEM:F12 (Life Tech.), supplemented with 1% N2 (Life Tech.), 1% non-essential amino acids (BioInd.), 1% Glutamax (Life Tech.), and 100 μg/ml Primocin (InvivoGen). N2/B27 Medium was a 1:1 mixture of N2 and B27 media. B27 medium was composed of Neurobasal (Life Tech.), supplemented with 1% B27 (Life Tech.), 1% non-essential amino acids (BioInd.), 1% Glutamax (Life Tech.), and 100 μg/ml Primocin (InvivoGen). Differentiation was carried out three times for each hESC line, cells were analyzed between days 40 and 160.

### Electrophysiology and pharmacology

Recordings were conducted as previously described (Telias et al., 2013, 2014, 2015a). In brief, neurons differentiated on glass coverslips were transferred to a recording chamber in standard recording medium, containing (in mM):10 HEPES, 4 KCl, 2 CaCl_2_, 1 MgCl_2_, 139 NaCl, 10 D-glucose (340 mOsm, pH 7.4). Cells were patch-clamped with pipettes containing (in mM) 136 K-gluconate, 10 KCl, 5 NaCl,10 HEPES, 0.1 EGTA, 0.3 Na-GTP, 1 Mg-ATP, and 5 phosphocreatine, pH 7.2 (pipette tip resistance was 5–8 MΩ). Membrane potential was held at −60 mV. GABA (2 mM in the pressure pipette, tip diameter 2–4 μm) was applied within 10 μm from the recorded neuron for a duration of 50 ms. Signals were amplified with a Multiclamp700B amplifier and recorded with Clampex 9.2 software (Axon Instruments). Bicuculline (Sigma) was applied at a concentration of 20 μM into the recording bath.

### Quantitative real time PCR

Relative transcription levels were analyzed using quantitative Real Time PCR (qRT-PCR) on cDNA reversed transcribed from RNA samples. RNA extracted from cells (RNAeasy Mini Kit–Qiagen), was stored at −80°C with RNAse Inhibitor (Roche) and DNAseI (Roche). RNA (100 ng) was reversed transcribed with Super Script III RT-PCR kit (Invitrogen). Quantitative Real Time PCR (qRT-PCR) was performed using SYBR Green (ABgene). Cycling and analysis were performed using Rotor Gene 6000 Series (Corbett) and its complementary analysis software. The house keeping gene *GAPDH* was used as a control for ΔΔCt analysis of results. All qRT-PCR assays included Non-Template Control (NTC), and adult human-FXS white blood cells. Oligo DNAs (custom made primers) were purchased from Sigma, and their sequences are as follows: *GAPDH*, Fwd: AGCCACATC GCTCAGACACC, Rev: ATACGACCAAATCCGTTGACTC; *Gephyrin*, Fwd: ATGATCCTTACTAACCACGACCA, Rev: AGATTTATCCCACTGCGGTCTT; *GABA-A*α*1*, Fwd: GGAAGAAGCTATGGACAGCCG, Rev: AATCCTGGTCTCAGGCGATTG; *GABA-A*α*2*, Fwd: GCCAATCAATCGGAAAGGAGAC, Rev: TTCCCATCCCAAGTCCATCCTC; *GABA-A*β*2*, Fwd: TGCCTGATACCTATTTCCTGAACG, Rev: GATTCCTAATGCCACCCTTGC; *GABA-A*γ*2S*, Fwd: TGCACACTCATTGTCGTCCTATCCTGG, Rev: TTAAACAGGCAGAAGGCAGTGGGG; *GABA-A*δ, Fwd: AGGACATCGTCTACTACTGGTCGGAGAG, Rev: TCGGCGTTGAAATGAGCA AAGG.

### Immunofluorescence

Immunostaining was performed as described previously (Telias et al., 2013, 2015b). Briefly, cells were fixed for 15 min at room temperature (RT) using Cytofix (BD Biosciences) and washed with PBS. Primary antibodies were incubated overnight at 4°C, in a PBS solution containing 2.5% BSA and 0.1% Triton. Staining with secondary antibody was performed for 1 h at RT in the dark. Primary antibodies, mouse anti-human TUJ1 (Millipore), and rabbit anti-human GAD 65 (Santa Cruz), were detected using sheep anti-mouse Cy2-conjugated or goat anti-rabbit Cy3-conjugated secondary antibodies (Jackson Laboratories). Nuclei were stained with DAPI (Sigma-Aldrich). Images were taken using a Zeiss LSM 700 confocal microscope.

### Data analysis

Data from patch clamp recordings were subjected to a 500-Hz low-pass filter and analyzed using Clampfit-9 and SigmaPlot. Statistical analysis was performed using SigmaPlot and online GraphPad QuickCalcs (http://www.graphpad.com/quickcalcs). Difference level of <0.05 was considered significant.

## Results

### Responses to GABA in neurons differentiated *in-vitro* from human embryonic stem cells

**Figure 1:**
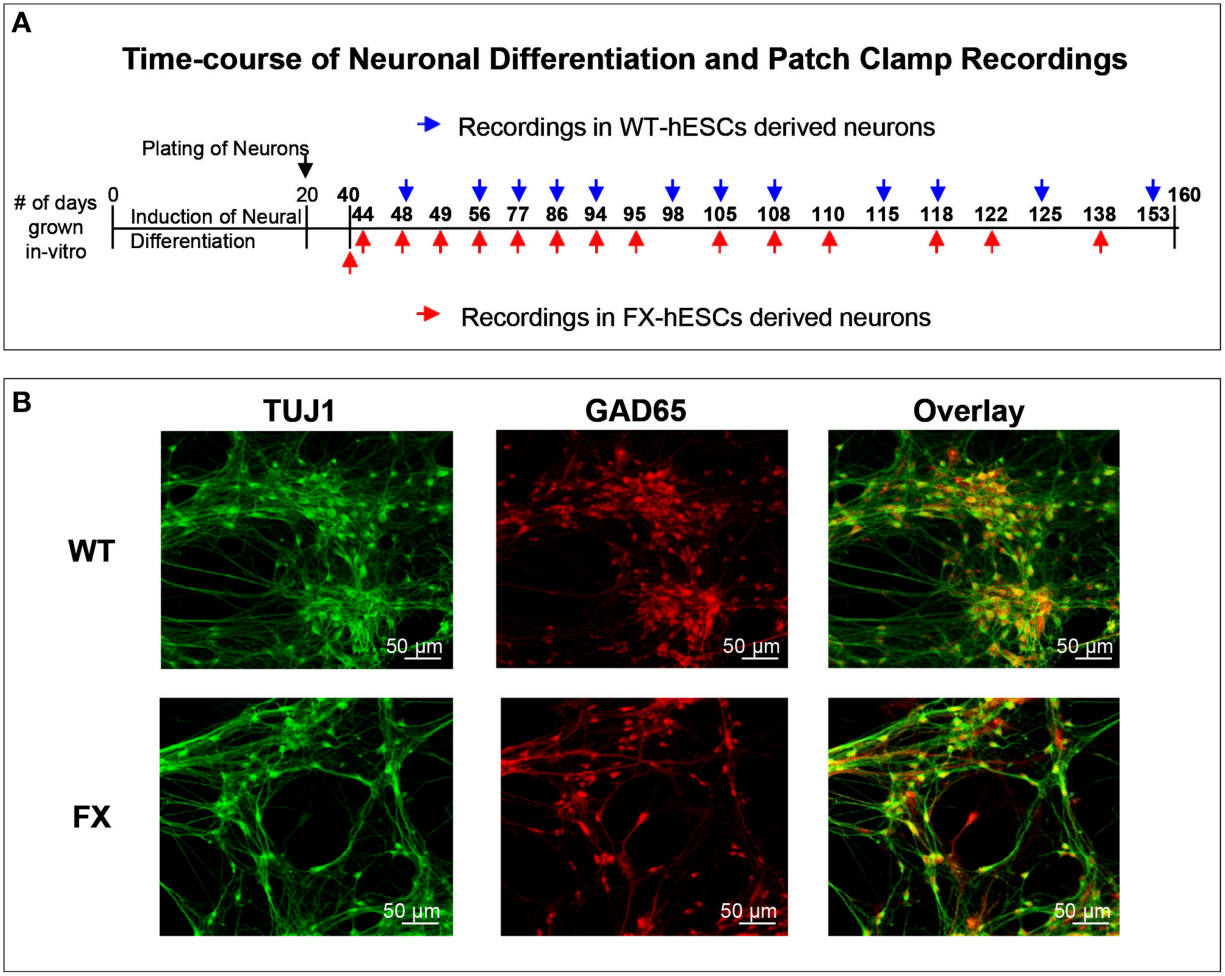
**(A)** Outline of the time-course of neuronal differentiation and the time-points at which electrophysiological recordings were carried out in WT (blue) and FX (red) human embryonic stem cells-derived neurons. **(B)** Immunofluorescence images for cellular localization of TUJ1 (green, left), GAD65 (red, middle) and their overlay (yellow, right); in WT (top row), and FX (bottom row) 40-days old neurons. Scale is 50 μm.

**Figure 2:**
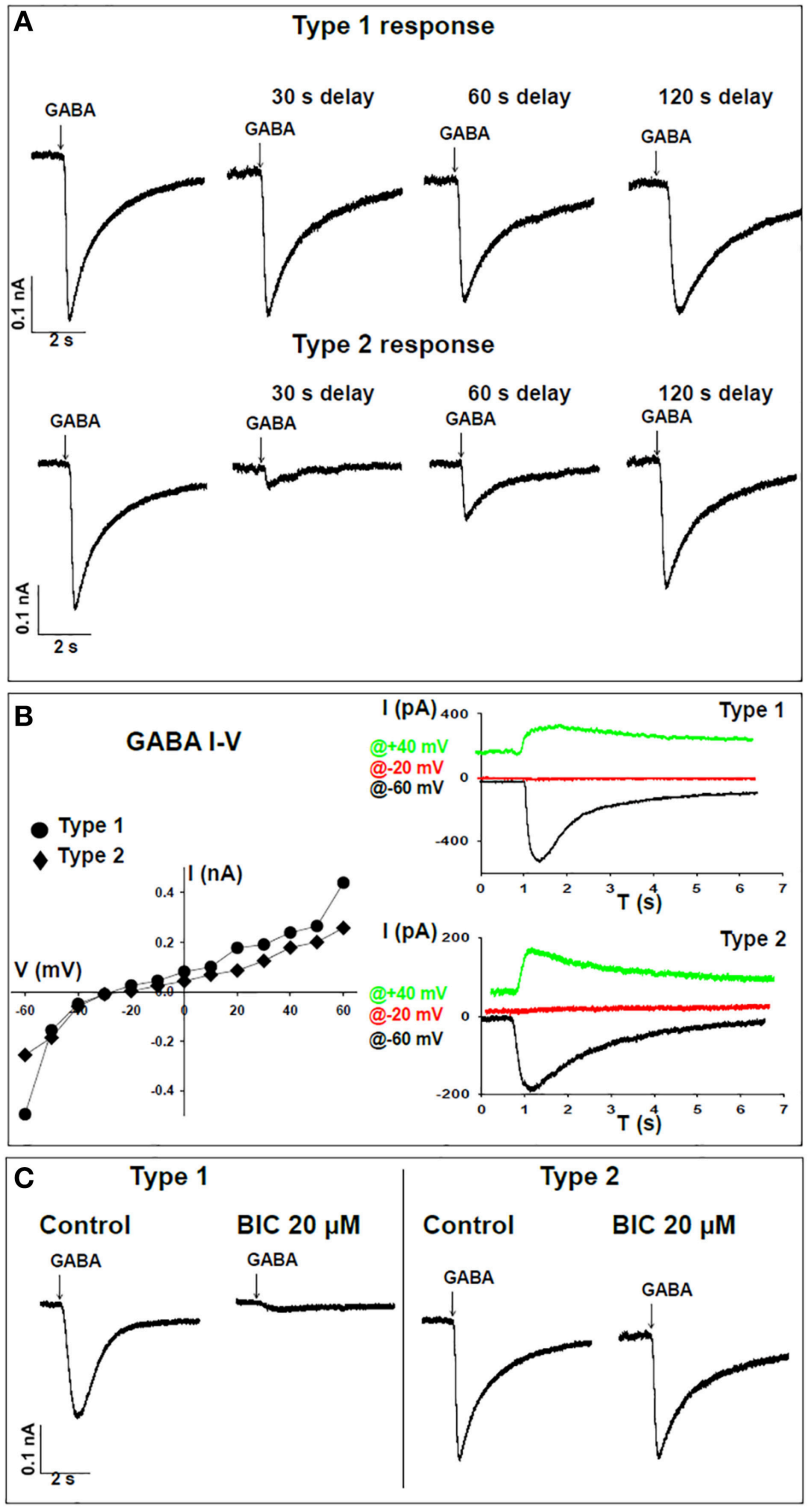
**Time-dependent responses of WT neurons to GABA**. **(A)** Representative traces of voltage-clamp recordings for time-dependent responses to GABA demonstrating “type 1” (top) and “type 2” (bottom) patterns. Cells were held at -60 mV and 2 mM GABA was applied by pressure from an adjacent pipette. Recordings were taken for the first puff and following consecutive delays of 30, 60, and 120 s. **(B) Left** panel, I-V curve for type 1 (circles) and type 2 (diamonds) GABA responses in single neurons. **Right** panel, representative traces corresponding to the same neuron shown on the left. Traces are sown for recordings at −60 mV (black), −20 mV (red), and +40 mV (green). **(C)** Representative traces of voltage-clamp recordings showing sensitivity to 20 μM bicuculline (BIC) in type 1 (WT *n* = 6, FX *n* = 1) and lack of sensitivity to BIC in type 2 (WT *n* = 5, FX *n* = 8) responses to GABA.

The responses of FX and WT neurons to GABA were examined between days 40 and 160 of *in-vitro* neural differentiation, carried out three times in three FX-hESC lines (designated FX1, FX2, and FX3) and one WT-hESC line (see Section Materials and Methods and Figure 1A). As we previously reported, both WT- and FX-derived neurons express the neuronal proteins NeuN, CaMK-II, TUJ1, and MAP2, but FX neurons do not express *FMR1* or FMRP (Telias et al., 2015a). Here we show that already at day 40 of differentiation, both WT and FX neurons, express co-localized GAD65 (glutamic acid decarboxylase 65) and TUJ1, indicating that these neurons have the enzymatic machinery necessary for producing and secreting GABA (Figure 1B). From these immunostaining assays we could estimate that >~90% of TUJ1-positive cells were also positively stained for GAD65. We have already shown that critical differences in firing patterns and synaptic activity exist between FX and WT neurons (including impaired action potential firing and reduced spontaneous synaptic activity) during the same time frame of differentiation considered in the present study (Telias et al., 2015a). In the present study we analyzed the electrophysiological responses of WT and FX neurons to topical application of GABA. At several time-points during differentiation (Figure 1A—blue and red arrows), we carried-out patch clamp recordings on WT and FX-hESC derived neurons. Voltage-clamped cells, held at −60 mV were exposed to a 50-ms, 2 mM GABA applied through a pressure pipette. Two distinct responses to GABA were found in both WT and FX neurons, tentatively designated herein as “type 1” and “type 2.” A total of 30 WT neurons were recorded as compared to 35 FX neurons (FX1 *n* = 11, FX2 *n* = 9, FX3 *n* = 15), in three differentiation experiments (Figures 2A,B). Type 1 responses were characterized as persistent and non-desensitized inward currents to successive pulse application of GABA. In contrast, type 2 responses showed time-dependent desensitization of reactivity to GABA, such that a consecutive GABA pulse applied ~1 s later resulted in no response, whereas 30 s later a ~80% reduction in the peak amplitude of the recorded current was still evident, and a full recovery was found more than 120 s after the first response (Figure 2A). However, both type 1 and 2 responses showed similar reversal potentials (Er), between −30 and −20 mV at an equivalent age (Figure 2B). In addition, the two types differed in their sensitivity to the GABA-A receptor antagonist bicuculline (BIC, at 20 μM; Figure 2C). In type 1 responses, BIC abolished GABA induced currents in all cells tested (WT *n* = 6, FX *n* = 1), while type 2 responses were insensitive to BIC (WT *n* = 5, FX *n* = 8). It is important to note that hyperpolarization of cells to −100 mV speeded up recovery from desensitization by ~50% in both WT and FX neurons (WT *n* = 3, FX *n* = 4), and that none of the cells analyzed, WT or FX, responded to pressure application of the GABA-B agonist baclofen (10–20 μM; WT *n* = 7, FX *n* = 6), indicating that the responses are of GABA-A type.

### Age distribution of type1 and type2 in WT and FX

**Figure 3:**
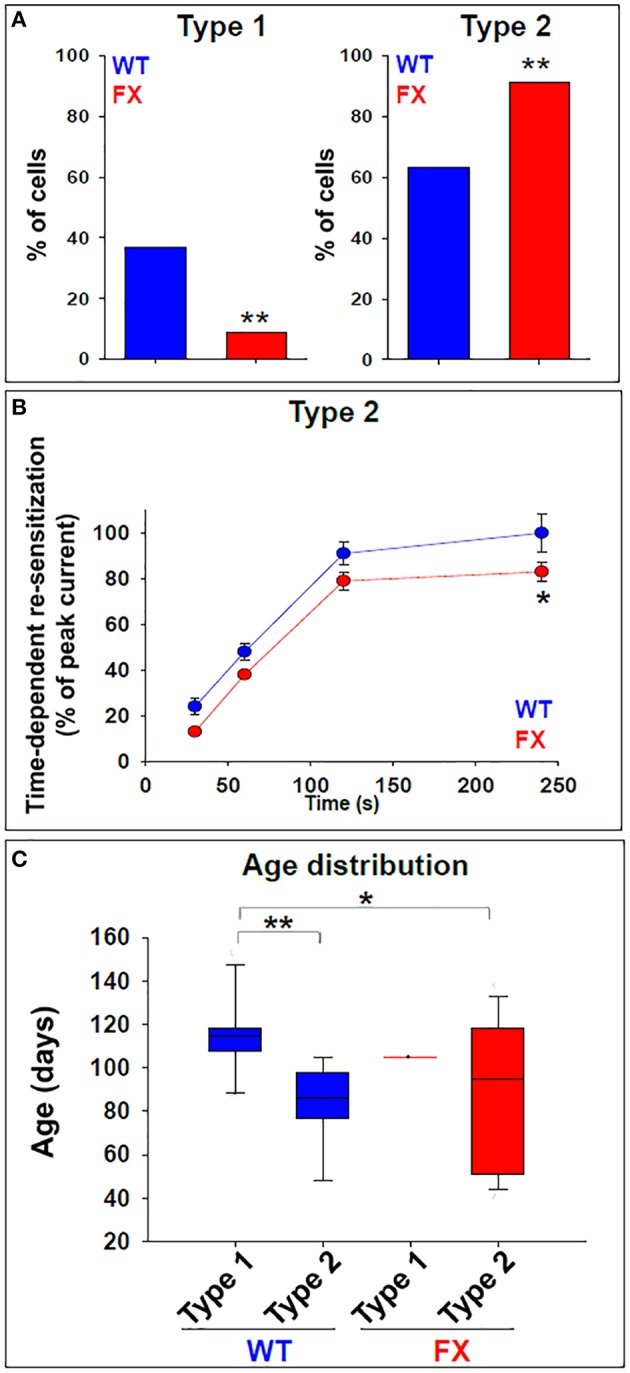
**GABA responses in WT and FX neurons. (A)** Total incidence of type 1 and type 2 responses to GABA in WT (blue) and FX (red) neurons. Values are the percent of cells in each category out of the total number of cells examined. WT *n* = 30, FX *n* = 35 (FX1 *n* = 11, FX2 *n* = 9, FX3 *n* = 15). ^**^p < 0.01, Chi-square test. **(B)** Quantification of time-dependent re-sensitization in type 2 responses, in WT (blue) and FX (red) neurons. Values are presented as percent of the peak current obtained with the first GABA pulse (before desensitization). WT *n* = 30, FX *n* = 35 (FX1 *n* = 11, FX2 *n* = 9, FX3 *n* = 15). Values are mean ± SEM. ^*^p < 0.05, *t*-test. **(C)** Box plot showing distribution of type 1 and type 2 responses in relation to neuronal *in-vitro* age following differentiation induction, in WT (blue) and FX (red) neurons. WT *n* = 30, FX *n* = 35 (FX1 *n* = 11, FX2 *n* = 9, FX3 *n* = 15). ^*^p < 0.05, ^**^p < 0.01, Chi-square test. See also Table 1 for details.

Next, we analyzed the frequency distribution of type 1 vs. type 2 responses, in FX and WT neurons. Our results show that 37% of WT neurons (*n* = 11 of 30) were classified as having type 1 responses, while 63% (*n* = 19 of 30) were classified as type 2 (Figure 3A). In contrast, in FX neurons, only 9% (*n* = 3 of 35, all 3 from FX3 line only) were classified as type 1, while 91% (*n* = 32 of 35) were of type 2. This bias of FX neurons toward type 2 responses was statistically significant (*p* < 0.01). Furthermore, within type 2 responses, time-dependent re-sensitization to GABA was significantly decreased in FX neurons as compared to WT (Figure 3B), suggesting that FX neurons have a higher tendency to a type 2-like response, with increased desensitization in reaction to GABA and reduced capability for re-sensitization. More importantly, the distribution of both types of response was age-dependent in WT but not in FX neurons (Figure 3C). Type 1-WT neurons were older, with an *in-vitro* age ranging from 86 to 153 days, while type 2-WT neurons were younger, with an *in-vitro* age ranging from 48 to 105 days (Table 1). This difference in age distribution between type 1 and type 2 response in WT was statistically significant (*p* < 0.05). In contrast, no age-dependency was found in FX neurons. Only three cells were classified as type 1-FX, which were all 105 days old and derived from line FX3 only. In contrast, type 2-FX neurons showed an age distribution ranging from day 40 to 138, which was significantly different from type 1-WT, but not from type 2-WT or type 1-FX (Table 1). Therefore, we conclude that type 2 response to GABA corresponds to an immature neuronal phenotype, which develops into the mature type 1 response. The strongly-biased distribution of FX neurons toward type 2 suggests that *FMR1* downregulation induced a significant delay in the transition from an immature to a mature neuronal phenotype, highlighting the important role played by FMRP in human embryonic neuro-development in general, and in the process of “GABA maturation” in particular. In our recently published study on the electrophysiological deficiencies of human FX neurons derived from hESCs (Telias et al., 2015a), we showed that lack of spontaneous synaptic activity in FX cells was rescued by co-culture with rat hippocampal neurons (Figure S1A). However, co-culture did not reverse the type 2 response to GABA in FX neurons (Figure S1B), further emphasizing the idea that FMRP may exert a direct effect on GABA-A receptor composition.

**Table 1:** **Full data for age distribution**.

**WT**				**FX**			
**Type 1**		**Type 2**		**Type 1**		**Type 2**	
**No. of cells**	**Age (days)**	**No. of cells**	**Age (days)**	**No. of cells**	**Age (days)**	**No. of cells**	**Age (days)**
1	86	1	48	1	105	1	40
2	98	2	48	2	105	2	40
3	108	3	48	3	105	3	44
4	108	4	56			4	44
5	115	5	77			5	44
6	115	6	77			6	48
7	118	7	86			7	49
8	118	8	86			8	49
9	118	9	86			9	56
10	125	10	86			10	56
11	153	11	94			11	77
		12	94			12	86
		13	94			13	94
		14	95			14	94
		15	98			15	94
		16	98			16	95
		17	105			17	95
		18	105			18	95
		19	108			19	108
						20	108
						21	108
						22	110
						23	110
						24	118
						25	118
						26	118
						27	118
						28	122
						29	122
						30	138
						31	138
						32	138
Mean	114.73	Mean	83.63	Mean	105	Mean	89.81
Sem	5.04	Sem	4.54	Sem	0	Sem	5.76
*n*	11	*n*	19	*n*	3	*n*	32

*Full data corresponding to Figure 3C.The results for two-tailed p-values obtained from unpaired t-tests are as follows: WT, Type 1 vs. Type 2: 0.000152; FX, Type 1 vs. Type 2: 0.432; Type 1, WT vs. FX: 0.347; Type 2, WT vs. FX: 0.459; Type 1-WT vs. Type 2-FX: 0.0203; Type 2-WT vs. Type 1-FX: 0.082. There was no statistically significant difference (p > 0.05) in the age range of WT neurons ([48–153], n = 30) as compared to the age range of FX neurons ([40–138], n = 35)*.

### Expression of GABA-A receptor subunits in WT and FX neurons

**Figure 4:**
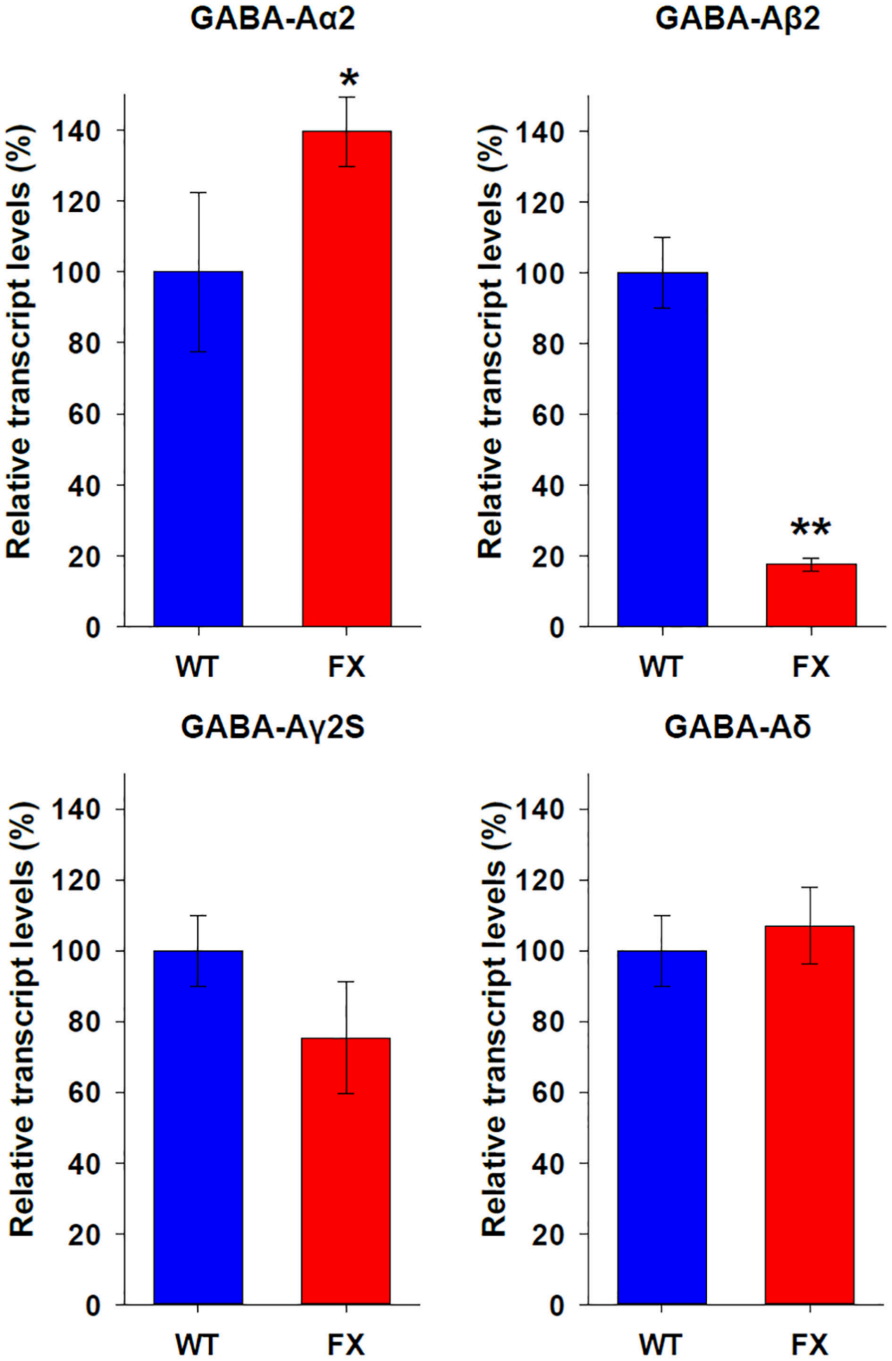
**Expression of GABA-A receptor subunits in WT and FX neurons**. Quantitative Real Time PCR analysis of relative transcript levels for the *GABA-A* receptor subunits: α*2*, β*2*, γ*2S*, and δ. Analysis was conducted on RNA isolated from age 120 to 130 days WT (blue) neurons in 2 different experiments, and from all three FX lines (red) in one experiment for each line; in triplicates. Values were normalized to *GAPDH* (ΔΔCt) and are shown as mean ± SEM (% of control). ^*^p < 0.05; ^**^p < 0.01; *t-*test.

In order to elucidate the molecular mechanism responsible for the biased tendency of FX neurons toward type 2 responses, we analyzed the expression of *Gephyrin* (which is specifically expressed in GABAergic neurons) and several subunits of the GABA-A receptor. RNA was extracted from WT and FX neurons at 120–130 days of differentiation, when most WT neurons respond to GABA as type 1, but FX neurons are characterized as type 2 (Figure 3C). We found that WT and FX neurons express similar levels of *Gephyrin* and do not express *GABA-A*α*1* subunit (data not shown). In contrast, FX neurons expressed significantly higher levels of *GABA-A*α*2* subunit (WT = 100 ± 22.5%; FX = 139.6 ± 9.8%; *p* < 0.05), and significantly lower levels of *GABA-A*β*2* subunit (WT = 100 ± 10%; FX = 17.7 ± 1.8%; *p* < 0.01; Figure 4). Non-significant differences in expression were found in *GABA-A*γ*2S* (WT = 100 ± 9.3%; FX = 75.3 ± 15.8%; *p* > 0.05), and in *GABA-A*δ subunit (WT = 100 ± 11.5%; FX = 107.1 ± 10.8%; *p* > 0.05). These results suggest that the significant increase in the mRNA expression of α2 subunit and the robust reduction in the mRNA expression of β2 subunit in FX neurons could be responsible for the persistence of the immature type-2 response in FX lines, and hint to a possible developmentally regulatory role for FMRP in the expression of specific GABA-A subunits.

**Figure 5:**
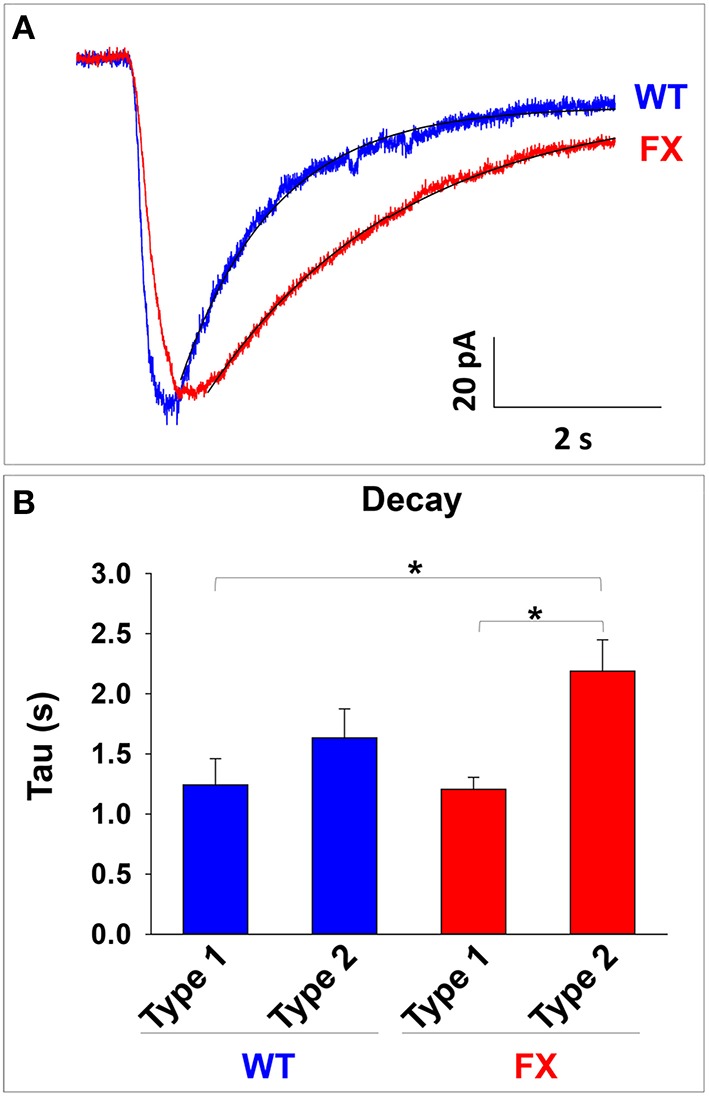
**Analysis of GABA response decay in WT and FX neurons. (A)** Representative traces of GABA responses and the exponential curve fit used to calculate the value of the decay. A trace from a type 1 response in WT is shown in blue and a trace from a type 2 response in FX is shown in red. Fit curves are shown in black. **(B)** Measurement of the decay (Tau) in WT and FX neurons, for type 1 and type 2 responses. Values are shown as mean ± SEM. ^*^p < 0.01, ANOVA.

An increase in α*2* expression has been linked to slower decay time in GABA-induced currents both *in-vivo* and *in-vitro* (Okada et al., 2000; Dixon et al., 2014). Therefore, we analyzed the value of tau in GABA responses in WT and FX neurons (Figure 5A). Our analysis shows a statistically significant increase in tau (i.e., slower mono-exponential decay) in FX neurons displaying type 2 responses as compared to type 1 responses in WT and FX (both *p* < 0.01; Figure 5B). Tau had a non-significant tendency to increase in type 2 responses in WT neurons as compared to type 1-WT (*p* = 0.12) or as compared to type 1-FX (*p* = 0.06), and to decrease as compared to type 2-FX (*p* = 0.07). These results suggest that an increase in α*2* mRNA in FX cells could be responsible for the immature response to GABA observed in the vast majority of FX neurons.

## Discussion

In this study we examined the effect of the *FMR1* mutation on the functional and molecular aspects of the GABAergic system in human neurons. We have shown that WT neurons obtained by *in-vitro* neural differentiation of hESCs undergo an age-dependent phase in which they exhibit a non-classical response to GABA, characterized by time-dependent desensitization and lack of sensitivity to GABA-A antagonist, bicuculline. We show that, while this immature response is transient in WT neurons, it is persistent across FX lines and throughout development. In order to unveil the molecular mechanism behind this phenomenon, we analyzed the transcription of specific *GABA-A* receptor subunits and found an increase in the mRNA expression of the α*2* subunit and a robust reduction in the mRNA expression of the β*2* subunit. These changes in the expression of *GABA-A* receptor subunits might be associated with an increase in GABA-response decay time. Our results suggest a role for FMRP in regulating the maturation of GABAergic neurotransmission, characterized here for the first time in human neurons.

### Abnormal GABAergic transmission associated with Fragile X neurodevelopment

It has been previously established that GABA-A receptors are under-expressed in the hippocampus and cortex of *fmr1*^−∕−^ mice (D'Hulst and Kooy, 2009; Braat and Kooy, 2015). Most importantly, it has been demonstrated that FMRP directly binds mRNA of several GABA-A receptor subunits (Braat et al., 2015). GABAergic transmission plays a crucial role during neurodevelopment, in which it switches from excitatory to inhibitory action due to changes in the intracellular chloride concentration, affecting the development of neurons at various levels, including their proliferation, migration, and dendritic arborization (Ben-Ari et al., 2012). It has been recently reported that in *fmr1*^−∕−^ mice, this developmental switch from excitation to inhibition is delayed (He et al., 2014). Indeed, lack of FMRP in developing cortical neurons increases the expression of the Na^+^-K^+^-Cl^−^ co-transporter, delaying the hyperpolarization of Cl^−^ reversal potential. In this study, we have uncovered two distinct types of responses to GABA in human neurons: while some of the neurons showed the expected response to GABA (including consecutive responses to repeated exposures, which were abolished by bicuculline), other neurons responded with an atypical time-dependent desensitization to consecutive GABA applications and complete insensitivity to bicuculline. More importantly, we have shown the relevance of this specific response in the context of human neurogenesis and FXS: while this atypical response eventually disappears with age in control neurons, it persists in FX neurons. These results suggest a new mechanism for the developmental delays and cellular abnormalities observed during neurodevelopment of FX-hESCS, concomitant with gradual downregulation of *FMR1*.

Desensitization of GABA-A receptors has already been described in cultured hippocampal rodent neurons (Segal, 1993; Jones and Westbrook, 1995). Most importantly, such desensitization was associated with younger age (1–7 days-old) and disappeared with more advanced age (21–28 days-old; Segal, 1993). Expression assays in oocytes and HEK cells have produced data indicating that subunit composition directly affects the desensitization properties of GABA-A receptors (Martinez-Torres et al., 2000; Bianchi and MacDonald, 2002; Chang et al., 2002). In general, desensitization of GABA-A plays an important role in regulating synaptic inhibition and neurotransmitter release. However, the role it plays during embryonic neurogenesis, a period when FMRP is critical, is less clear. Owens et al. found that early cortical neurons display less desensitization than more mature neurons, and that GABA-A receptors are activated in a paracrine mechanism which does not involve synaptic activation (Owens et al., 1999). These results suggest that GABA plays extra-synaptic roles during neuronal maturation that are not yet fully understood More importantly, the role FMRP could play in the kinetics of GABA-A desensitization has not been established yet. Our study provides the first evidence for an FMRP-dependent maturation of GABA responses in human neurons developing *in-vitro*.

### Abnormal expression of specific GABA-A subunits in FX human neurons

The functional analysis of GABA response in WT and FX *in-vitro* human neurons led us to conclude that subunits in the GABA-A receptor could be abnormally expressed in FX neurons. We found that the α1 subunit was not expressed in WT nor in FX neurons. Furthermore, there was no significant difference between WT and FX neurons in the expression of γ*2s* and δ subunits. However, a significant increase in α*2* expression and a robust reduction in the expression of β*2* subunit were found in FX neurons as compared to WT, which could potentially explain the immature phenotype observed in FX neurons. A similar response to GABA, showing desensitization and lack of reactivity to bicuculline (similar to “type 2”) was shown by Maddox et al. in normal human dorsal root ganglion cells (DRGs) from adult samples, but not in embryonic counterparts (Maddox et al., 2004). In that study, and ours, a correlation between neuronal immaturity and reduced expression of the *GABA-A*β*2* subunit was observed. It is important to note that both studies, Maddox et al. and the current report, analyzed mRNA levels of *GABA-A* subunits, rather than protein levels. Therefore, any conclusion on the molecular conformation of the actual receptor could be made only under the assumption that changes in transcription directly reflect changes in translation, which might not be necessarily so. Taken together, these data suggest that FMRP plays an important role in the regulation of the development of the GABAergic system in early human neurons.

In rodents, an increase in α*2* expression has been linked with immature GABA responses characterized by slow decay in developing postnatal thalamic neurons (Okada et al., 2000). After maturation however, higher expression of α*1*, lower expression of α*2* and faster decay are observed in the same neurons, indicating that α*2* is probably responsible for slower decay. The same slow decay has been reported *in-vitro* in HEK cells following overexpression of α*2*, resulting in a ~10-fold slower decay for α*2* over α*1* (Dixon et al., 2014). In contrast, two different studies concluded that the slow decay component in GABA responses is a result of α*3* expression (Geracitano et al., 2012; Labrakakis et al., 2014). Here we suggest that, in accordance to previous studies, the increase in α*2* expression in FX neurons, which were predominantly “type-2,” could be responsible for the slower decay in GABA responses in these neurons. A hypothesis can be formulated by which FMRP plays a role in the downregulation of α*2* subunit during neuronal maturation. In FXS, where FMRP expression is lost, α*2* remains abnormally high, impairing maturation. However, in order to test this hypothesis, more experiments need to be conducted.

## Summary

The findings of the present study have never been reported in the developing brain of the *fmr1*^−∕−^ mouse, and highlight the potential significance of the FX-hESCs system in modeling FXS. However, more studies are necessary to further explore the role of FMRP in the GABAergic developmental switch and its contribution to the pathology of FXS.

## Author contributions

MT designed and performed the experiments, collected and analyzed the data, wrote the manuscript. MS designed the experiments, provided materials and funding, analyzed the data, wrote edited the manuscript. DB designed the experiments, provided materials and funding, analyzed the data, wrote and edited the manuscript.

## Funding

This study was supported by the National Network of Excellency (NNE) in Neuroscience from TEVA Pharmaceuticals Ltd and by a grant from the Israel Ministry of Health.

### Conflict of interest statement

The authors declare that the research was conducted in the absence of any commercial or financial relationships that could be construed as a potential conflict of interest. The reviewer LD and handling Editor declared a current collaboration and the handling Editor states that the process nevertheless met the standards of a fair and objective review.
