# Supplementary figures and images for: Immature Responses to GABA in Fragile X Neurons Derived from Human Embryonic Stem Cells
Source: Front Cell Neurosci. 2016 May 12;10:121. doi: 10.3389/fncel.2016.00121 (PMC4864171; doi:10.3389/fncel.2016.00121)

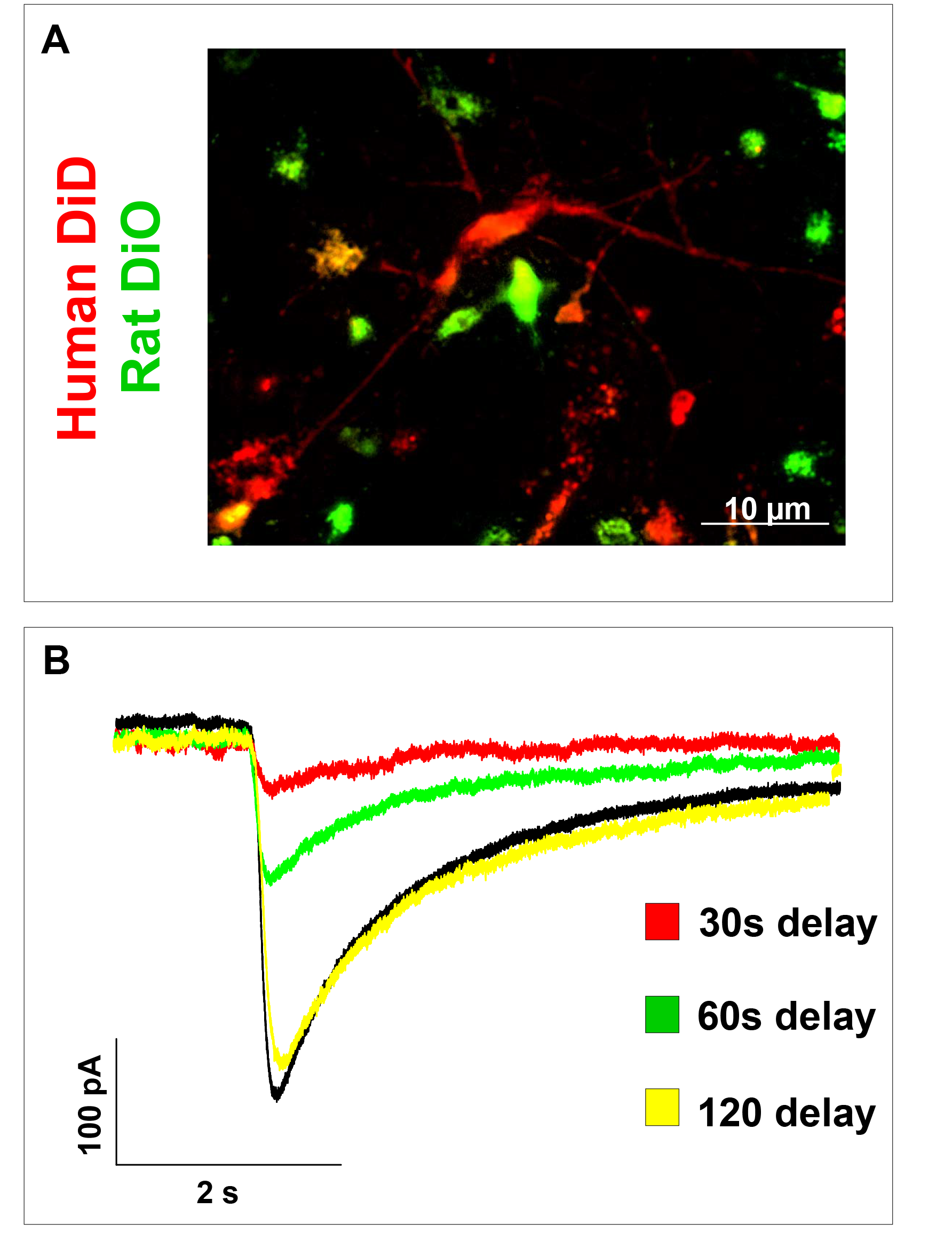

Supplement: Supplementary file 1 [file Image1.TIF]
