# Supplementary material for: Immature Responses to GABA in Fragile X Neurons Derived from Human Embryonic Stem Cells
Source: Front Cell Neurosci. 2016 May 12;10:121. doi: 10.3389/fncel.2016.00121 (PMC4864171; doi:10.3389/fncel.2016.00121)
Supplement: Supplementary file 2 [file DataSheet1.DOCX]

Legend to Supplementary Figure 1.

**Figure S1. GABA responses in co-culture.**

**(A)** Representative image of a co-culture assay including FX neurons derived from human embryonic stem cells stained with DiD (red) and adult rat hippocampal neurons stained with DiO (green). Scale bar is 10 µm.

**(B)** Representative traces showing time-dependent desensitization to consecutive GABA pulses in a human FX neuron co-cultured with rat hippocampal neurons, following the first pulse (in black), and after 30 (red), 60 (green) and 120 seconds (yellow). This experiment was carried out in a total of 4 cells.
